# Damage Analysis of Segmental Dry Joint Full-Scale Prestressed Cap Beam Based on Distributed Optical Fiber Sensing

**DOI:** 10.3390/s23073781

**Published:** 2023-04-06

**Authors:** Duo Liu, Shengtao Li, Joan R. Casas, Xudong Chen, Yangyang Sun

**Affiliations:** 1 The State Key Laboratory on Safety and Health of In-Service Long-Span Bridges, JSTI Group, Nanjing 210019, China; 2 College of Civil and Transportation Engineering, Hohai University, Nanjing 210098, China; 3 Department of Civil and Environmental Engineering, Technical University of Catalunya, UPC-BarcelonaTech, Campus Nord, Calle Jordi Girona 1-3, 08034 Barcelona, Spain; 4 College of Defense Engineering, PLA Army Engineering University, Nanjing 210014, China

**Keywords:** prefabricated bent cap, shear key, dry joint, strain state analysis, distributed optical fiber sensors

## Abstract

Distributed fiber optic sensors (DFOS) can detect structural cracks and structural deformation with high accuracy and wide measurement range. This study monitors the segmental prestressed bent cap, assembled with a large key dry joint, based on optical fiber technology, and it allows the comparison of its damaging process with that of a monolithic cast in place counterpart. The obtained results, comprising cross-section strain distributions, longitudinal strain profiles, neutral axis location, crack pattern, and the damage process, show that the DFOS technology can be successfully used to analyze the complex working stress state of the segmental beam with shear key joints, both in the elastic range and at the ultimate load, and to successfully identify the changing characteristics of the stress state of the segmental capping beam model when elastic beam theory no longer applies. The DFOS data confirm that the shear key joint, as the weak point of the segmental cap beam, results in the high stress concentration area, and the damage rate is higher than that of the cast-in-place beam. The accurate monitoring by the DFOS allows for the realization that the damage occurs at the premature formation of a concentrated compression zone on the upper part of the shear key.

## 1. Introduction

Assessment of the structural damage status of modern urban bridges is crucial to ensure their safety and reliability, especially with the increasing traffic volume and aging infrastructure [1,2]. Traditional visual inspection methods have been widely used, but they are often time-consuming and may not effectively detect hidden damage. Moreover, traditional damage assessment methods often only qualitatively describe the structural damage, while lacking accurate quantification of the damage level [3]. Therefore, more accurate and reliable structural damage assessment methods are needed to meet the needs of modern urban bridge construction and maintenance. 

In the last few years, several infrastructure sensing technologies have emerged, including ground-based laser scanning [4], unmanned aerial vehicle imaging [5], and distributed fiber optic sensing (DFOS) [6,7]. Among these, DFOS has proven to be a successful method for measuring distributed temperature and deformation, as well as detecting damage in civil infrastructure [8]. As DFOS technology continues to evolve, it is leading to improved spatial resolution, greater accuracy, and increased frequency, which enables the detection of local damage with greater precision and a transition from damage detection to damage quantification [9,10].

Optical frequency domain reflectometry (OFDR) is an optical sensing technology that can monitor small strains and temperature changes in real-time, making it a promising tool for health monitoring and structural damage assessment in engineering structures such as bridges [11,12,13,14]. OFDR technology utilizes optical fibers as sensors, and through the time-domain and frequency-domain information of the light signal, it achieves high-precision and high-sensitivity detection of small strains and temperature changes within the optical fibers [8,15]. OFDR technology has features such as high accuracy, large dynamic range, simultaneous multi-point detection, high spatial resolution, and online real-time monitoring, which meet the needs of health monitoring and structural damage assessment for engineering structures [16]. By installing fiber optic sensors in concrete structures, the monitoring of concrete and steel stress can be achieved [17,18], which can further evaluate the load-bearing capacity of concrete structures. 

Apart from structural concrete, other engineering materials were monitored by DFOS. Goodwin et al. [10] examined the structural behavior of glued laminated beams, using DFOS, under four-point bending. The research findings suggest that DFOS data can be utilized to measure the in-situ elastic modulus of wooden materials. Monsberger et al. [19] employed DFOS to assess the curvature and bending characteristics of grouted anchor bolts. The applicability of the fiber optic sensing system was validated through total station measurements. However, it is worth noting that not many studies have applied OFDR to evaluate the damage in concrete bridge components [20,21], and the quantitative analyses and predictions of structural damage in concrete structures, using fiber optic monitoring, still present challenges that require further development of data processing and analysis methods [22,23]. This lack of research is even more evident in the case of precast segmental concrete elements, where the existence of dry construction joints in the final structure can be challenging for the optical fibers crossing them or deployed in their vicinity. To the authors’ knowledge, this is carried out in the present work for the first time. In addition, the influence of fiber optic coatings or bonding agents on sensing results in normal concrete structures is still not fully understood [24,25], and this ignorance, again, is further emphasized in the case of precast segmental structures. This study employs OFDR technology and different types of optical fibers to attempt non-destructive monitoring and structural damage analysis of large-scale cap beam in precast assembly concrete bridges, which may help to address these challenges.

Concrete bent cap (CBC), one of the key components in bridge structures, is commonly used to provide lateral stability to piers, as well as to carry the loads coming from the deck and transmit them to the pier columns and foundation [26]. However, due to the increasing traffic load and other factors, the concrete beam is susceptible to various mechanical and environmental damages, such as bending, shearing, and reinforcement corrosion, leading to structural damage and failure if remedial measures are not taken on time [27]. For this reason, accurate monitoring of the degradation process is a main deal. Additionally, the damages of CBC often occur in a small range and short dimension, making it difficult to monitor and evaluate effectively through visual inspection methods [28]. Furthermore, segmented assembly technology has become increasingly popular in bridge construction to meet the requirements of construction schedule and quality [29,30]. This application of segmental precast bent cap and on-site assembly makes the bridge construction process faster, more efficient, and of a higher quality. It should be noted that the failure behavior of precast segmental concrete bent cap (PSCBC) with dry joints is different from that of cast-in-situ concrete bent cap (CICBC) [31,32]. Due to the complexity of this bridge element, it is necessary to establish reliable monitoring and evaluation methods combining multiple tests results. However, there is a lack of large-scale experimental research [33]. The strain data obtained from current experimental analyses are constrained and mainly based on single-point measurement, which cannot sense the overall strain state of the structure and neglects invisible information about structural performance [34]. Moreover, the ultimate bearing capacity, stiffness, and shear resistance are the main objects that the experiments primarily concentrated on [35]. They overlooked the variations in stress state that occur throughout the entire loading process of the structure, and thus, they were unable to expose the underlying mechanisms of structural failure.

This study focuses on the structural behavior and damage process of large bridge CBC using two experimental models: segmental and cast-in-place. Under monotonic loading, the specimens were tested in the structural laboratory. The ODiSI 6000 series sensing platform was used to achieve a distributed sensing measurement with OFDR technology. Optical fibers with different coatings were attached to the surface of the beam to study the structural behavior of PSCBC and CICBC. By using an innovative structural analysis method based on DFOS, this paper tries to expose the response characteristics of PSCBC under bending–shearing actions. The applicability of different types of optical fibers for bridge structure damage monitoring was also explored. Finally, the strain data shown by DFOS were used to further explore the differences in stress state and failure mode between PSCBC and CICBC. This paper first introduces the test setup and fiber layout (Section 2), followed by the presentation and discussion of DFOS test results in the test (Section 3).The differences in damage characteristics between PSCBC and CICBC are also evaluated in Section 3 based on the DFOS results.

## 2. Experimental Details

### 2.1. Test Set-Up

**Figure 1:**
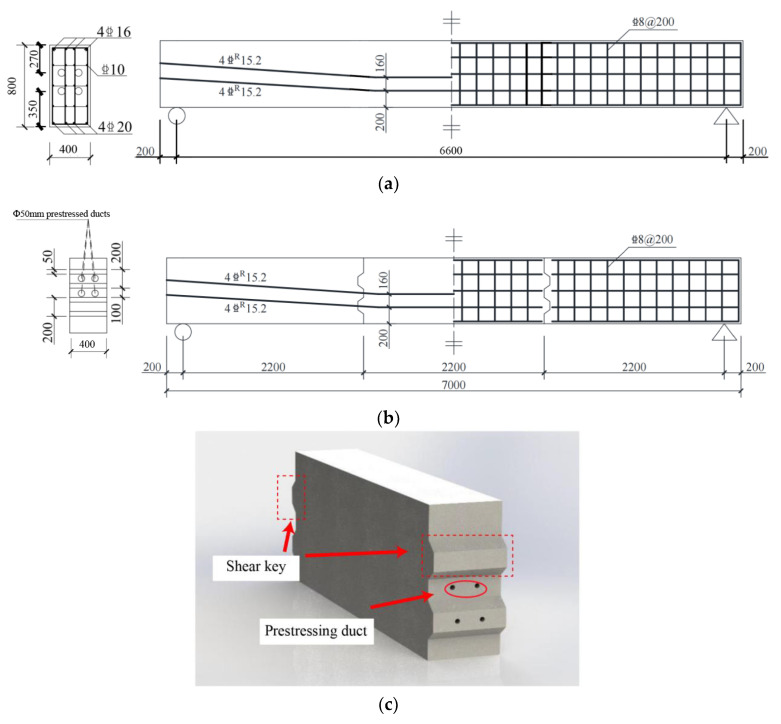
Reinforcement design and dimension of precast segmental beams and cast-in-place concrete beams: (**a**) cast-in-place beams; (**b**) precast segmental beams; (**c**) schematic diagram of a segment.

**Figure 2:**
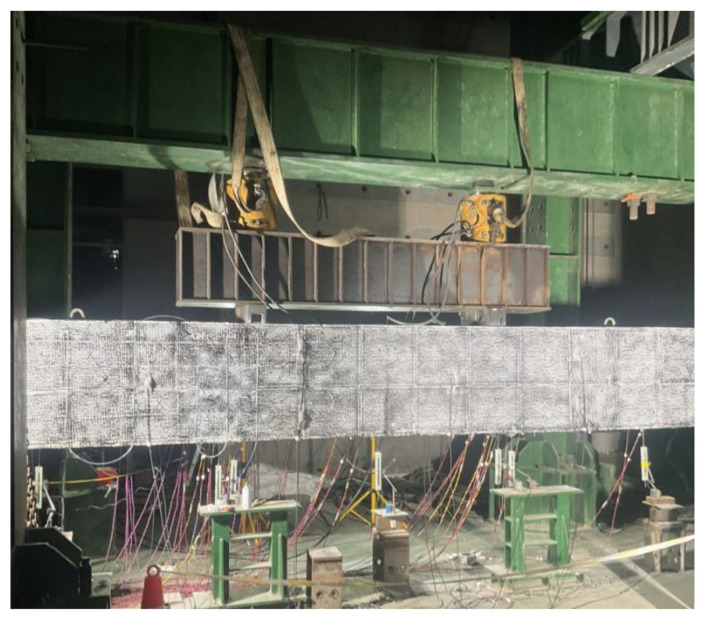
Laboratory test setup of precast segmental bent cap.

Following the structural design of the bent cap in the actual project (Nanjing 312 National Highway Reconstruction and Expansion Project), a simplified beam model with an appropriate cross-sectional shape was designed. The experimental concrete beam has a length of 7000 mm and a cross-sectional dimension of 400 mm width and 800 mm depth. The longitudinal reinforcement diameter for tension is 20 mm, and for compression, it is 16 mm. The design dimensions and reinforcement design of the cast-in-place beam are shown in Figure 1a. The support is 200 mm away from the beam end, with an effective span of 6600 mm. The dimensions and reinforcement of the precast segmental beam with large-keyed joints are shown in Figure 1b. The cross-sectional and reinforcement design of the cast-in-place beam and the precast beam are basically the same, but the precast beam is composed of three segments, as shown in Figure 1c. The joints between sections are assembled with a large keyed dry joint, and they are prestressed. The precast sections have reserved prestressing ducts, and the reinforcing steel bars are cut off at the joints. The concrete cover thickness (the outermost layer of steel bar protection) for all beams is 28 mm, and the rebar anchorage length is 200 mm, with a reinforcement ratio of 0.393%. According to the specifications and existing projects, the prestressed steel strands are tensioned to 70% of the yield stress (1395 MPa) to meet the average design compressive stress in the beam cross-section, which is 5.45 MPa. The actual photo of the testing setup is shown in Figure 2. A monotonic load is applied through and hydraulic actuator located at the top of the beam, simulating the actual stress state of the beam in normal operation. The four-point bending loading method is used, and the load is transmitted to the test beam through the upper distribution beam. The loading is carried out at a distance of 900 mm on both sides of the middle section. In addition, a large number of speckle points are painted on the surface of the beam, and digital images are collected during the test. By using the digital image correlation (DIC), the strain field can be obtained for comparison with the results of the fiber optic monitoring. 

### 2.2. Concentric Cable Configurations and Fiber Layout

**Figure 3:**
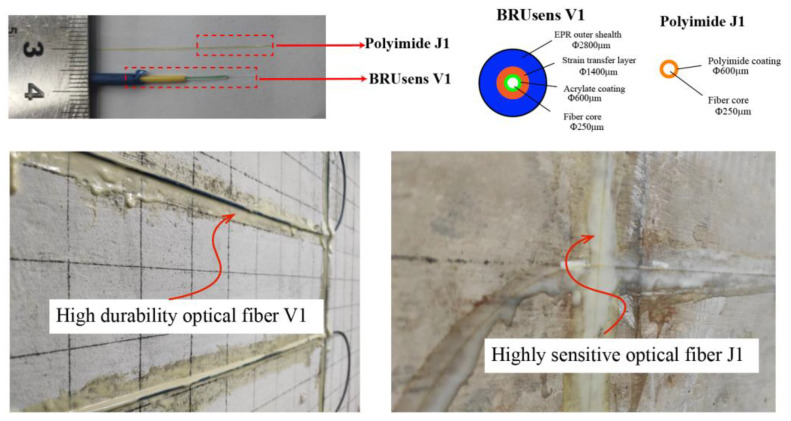
Field test setup of precast segmental bent cap.

The ODiSI 6000 Series from Luna Innovations Inc. (Roanoke, VA, USA) was used as interrogator for the DFOS. This system is based on the Rayleigh scattering of optical frequency-domain reflectometry (OFDR) technology. The light emitted by a light tunable laser source is divided into two paths: one works as the reference light, and the other one enters the optical fiber. On its way forward along the fiber, the Rayleigh scattering signal carries information on geometrical and physical characteristics of the fiber. The Rayleigh scattering signal is reflected back and interferes with the reference light. By correlating the recorded signal with the reference one, Rayleigh frequency shift can be obtained and correlated to strain and temperature changes along the fiber. If the fiber is bonded or embedded into a host substrate material, the strain in the material is measured by the fiber. Rayleigh scattering can achieve single-end measurement with high spatial resolution and measurement accuracy, despite these measurements being limited within the sensing range [8,15]. In our tests, the system adopted a spatial resolution of 1 cm and a measurement range of 100 m, providing real-time monitoring capabilities and allowing the efficient collection and analysis of data. In addition to its high performance, accuracy, and high spatial resolution, the key part also involves the selection of the optimal optical fiber and the best installation process. In the present work, two types of fiber optic cable configurations for the monotonic loading tests were selected. The first one, J1, is a polyimide-coated fiber. The fiber consists of a polyimide coating layer and an internal fiber core. The total diameter is 155 μm, consisting of core, cladding, and coating. The coating is only 15 μm. This type of fiber optic cable (Figure 3) is a high-performance fiber with high tensile strength and chemical stability. Due to its thin coating layer, it has excellent strain transfer performance and high sensitivity to structural strain changes, but it also has relatively poor maneuverability and durability due to its higher brittleness. The other type of fiber optic cable, “BRUsens V1”, has multiple coating layers, consisting of a 4-layer concentric structure of multiple buffering layers, strain transfer layers, and an EPR (ethylene propylene rubber) outer sheath with an outer diameter of 2800 μm (as shown in Figure 3). Multiple-layer coating gives the fiber optic cable very strong toughness, and the outer sheath has anti-corrosion properties, providing good protection to the optical fiber. Its high durability helps the fiber optic cable resist the effects of multiple external loads during the long-term monitoring of concrete structures. However, due to the thicker coating layer, the sensitivity performance is reduced. The performance of these fiber optic cables with very different characteristics were tested in previous studies, when deployed in monolithic concrete elements, to explore their usability in damage monitoring [21,35]. Therefore, in the present research, the objective was to test their performance when externally bonded to segmental concrete beam structures. The two fiber types were externally bonded to the concrete specimens, as shown in Figure 3, by using epoxy adhesives as the bonding agent.

**Figure 4:**
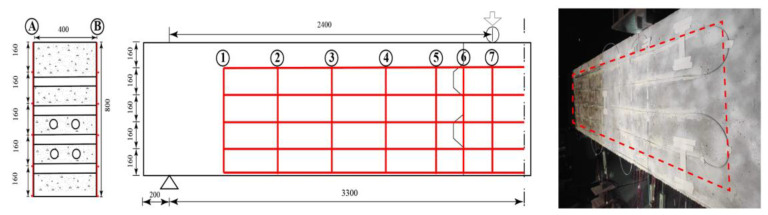
Schematic diagram and picture (side A of the beam showing fiber V1) of fiber optic strain monitoring layout on the cap beam.

In the test, the DFOS were installed as shown in Figure 4. In this way, continuous strain monitoring points were arranged on multiple cross-sections. There were seven different cross-sections recorded from the support end to the loading point direction. Sections 1–5 were evenly spaced at a distance of 400 mm. Additionally, the joints between the segments in the precast beam are critical points of the structure where high stress states can develop, so fiber optic cables were densely arranged near the joint between the beam segments, with a distance of 200 mm between Sections 5–7. Each fiber optic cable on each monitoring section was bonded from the bottom of the beam and extended to a length of 640 mm upwards. Although the monitoring system can achieve a resolution of up to 1 mm, the resolution of the fiber optic monitoring in this study was set to 1 cm, considering the sizes of the signal data and transmission rate. The DFOS system was installed on both sides of the beam, so there were 128 surface strain measuring points on each single cross-section in Sections 1–7. In addition, fiber optic cables were also installed longitudinally on the beam for monitoring crack development, with a total of five levels installed from top to bottom. Fiber optic cable V1 was bonded in lateral side A, and fiber optic cable J1 was bonded with a similar layout in the lateral side B (see Figure 4). It is worth noting that the implementation of DFOS in prestressed cap beam monitoring requires proper planning and execution. The installation of the optical fibers within the beam must be done carefully to ensure that they are not damaged during the construction process and that they are correctly positioned to provide accurate measurements. The system must also be calibrated correctly to ensure that the data obtained are accurate and reliable.

## 3. Fiber Optic Monitoring Results

### 3.1. Strain State Analysis at the Bottom of the Beam

**Figure 5:**
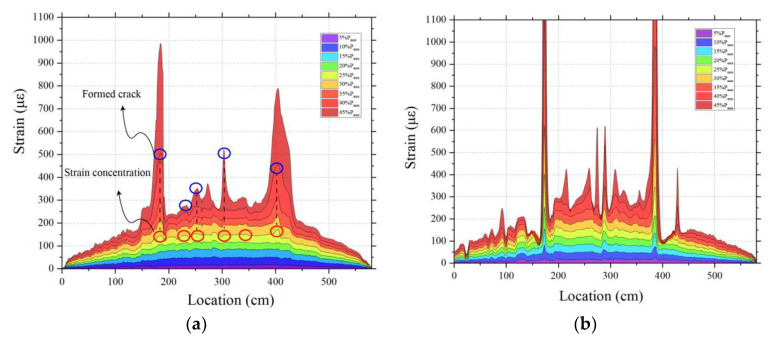
Strain profile at the bottom of precast prestressed bent cap under rising load: (**a**) Fiber V1 on side A; (**b**) Fiber J1 on Side B.

The DFOS strain profiles at the bottom of the PSCBC beam on both lateral sides, A and B, are shown in Figure 5. The strain distribution generally matched the theoretical distribution of a four-point bending loading state, where the strain increases linearly from the support end and, then, remains constant within the pure bending area. However, it should be noted that this theoretical behavior was only valid up to the load level of 20% of the ultimate load (Pmax). As the load further increased, strain peaks appeared in the strain profiles, indicating the initiation of cracks at the bottom of the beam. These peaks became higher and more pronounced with the increasing load, indicating that crack width continued to increase too. Several obvious strain peaks caused by cracks are identified by the blue circles in Figure 5a. The two strain peaks were most obvious near the shear keys on both sides of the beam, at the coordinates of 180 cm and 400 cm, and they appeared earliest in these regions. This indicated that the shear keys are the weak points of the precast segmental beam, and cracks would first occur in this area. The peak strain value at the crack location quantitatively reflects the crack width. It can be seen from the figure that the propagation rate of the shear key crack was much higher than that of other cracks, eventually forming the main crack. 

Another interesting finding was that the occurrence of cracks in the beam can be predicted by the fiber optic sensing system very early before they can be visually observed. As shown in Figure 5a, by observing the final crack peak (blue circle) and the early crack peak (red circle), the strain at the crack location exhibited an obvious strain gradient at an early stage, forming a small protrusion during the elastic stage, and gradually developing into a mature crack. The change in the strain indicating the initiation of a crack could be detected before 0.25P_max_, and as shown in Figure 5, it was first detected by the fiber J1. However, in the actual experiment, cracks were observed by the naked eye only at 35–40% of P_max_. This indicates that the damaging process of the beam can be warned, more accurately, by the fiber optic sensing system. On the other hand, the DFOS strain distribution at the same location along the section depth, on sides A and B of the beam, was not exactly the same. In fact, they showed significant differences. The DFOS on the opposite side at the same section depth had significantly different peak positions. This is a clear indication that the opening cracks inside the beam do not extend uniformly across the width of the beam. The most obvious difference in strain between sides A and B was at the shear key, as the side B of the shear key was much larger than on side A. In addition, the strain peak at the shear key on side B appeared much earlier. This might be due to the non-homogeneity of the concrete material, resulting in inconsistent damage distribution along the width of the beam, but it may also be due to the different shear stiffness of the cladding and coating materials present in the two fiber types, as well as the properties and thickness of the bonding layer [21,36].

**Figure 6:**
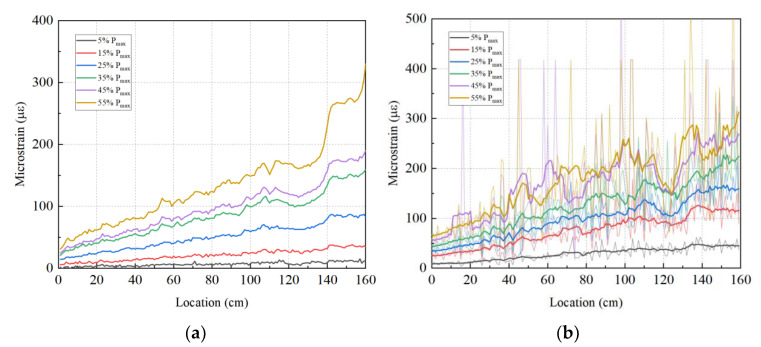
Strain at the left part of the bent cap under rising load: (**a**) Fiber V1; (**b**) Fiber J1.

Figure 6 shows a graph of the strain data obtained by the two types of optical fibers. The data show that the J1 fiber with a polyimide coating exhibits a larger local strain fluctuation. This fluctuation is relatively small in the low loading stages, and it gradually increases with the load. The data profile of the multi-coated fiber is smoother. Table 1 gives the average and variance of the strain increments between adjacent points for different fibers at different load levels. The strain increment parameter reflects the sensitivity of the fiber to structural strain. The mean value of the strain increment of J1 fiber is similar to that of V1 fiber, but the variance is much higher. When the load is 5% of Pmax, the mean value of strain increment at the adjacent point of J1 is 0.23 με, and the variance is 163.8 με^2^; when the load is 35% of Pmax, the mean value of the strain increment at the adjacent point of J1 is 0.82 με, and the variance is 3272 με^2^. The fluctuation in the measurement may be caused by the extremely thin optical cable structure of J1 fiber, and it has also been observed in other works [11,17,21]. This makes the roughness of the concrete surface uneven and easily generates high local strains under stress increases. Therefore, the strain data of J1 fiber needed to be post-processed by a moving average line or other similar techniques [37]. As shown in Figure 6b, after post-processing, the data profile of J1 and V1 fibers all show a straight line upwards, which is consistent with the deformation under four-point bending. However, the J1 fiber still reflects higher strain fluctuation. 

**Figure 7:**
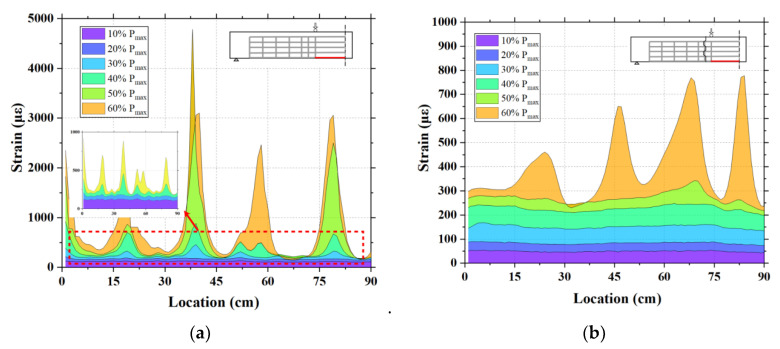
Strain profile at the bottom of the mid-span under the rising load, corresponding to the V1 sensor: (**a**) cast-in-place cap beams; (**b**) precast cap beams.

By comparing the strain distribution and crack development, differences in behavior between PSCBC beam and CICBC beam can also be observed. As shown in Figure 7a,b, at the same loading stage, although the crack spacing is roughly the same, the strain peak generated in the cast-in-place beam is much larger than the strain peak in the segmental beam. As shown in Figure 7a, the mid-span crack of the cast-in-place beam can be clearly identified at 30% of Pmax, with a maximum strain peak of about 450 microstrain, and the strain increases significantly at 40–50% of Pmax, with a maximum value of about 3000 με. However, for the precast segmental beam, the strain increase at mid-span is relatively gentle. When the load reaches 50% of Pmax, there is a clear strain peak, and when it reaches 60% of Pmax, the crack grows rapidly, with a maximum strain peak of about 800 microstrain. The difference in DFOS strain profiles indicates that there are significant differences in the damage and failure modes between the cast-in-place and the precast segmental beam. For the cast-in-place beam, the damage occurs at mid-span, where the main cracks develop. In the case of precast segmental beams, damage primarily manifests at the joint position; thus, cracks emerging at the mid-span do not constitute the primary cause of damage.

**Figure 8:**
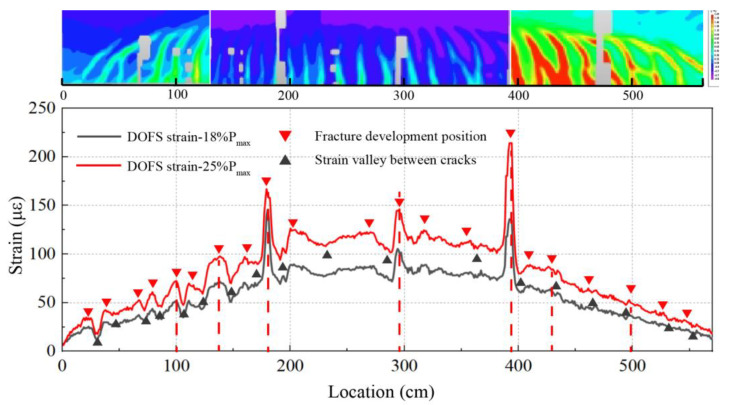
Crack location determined from the DFOS strain profile and compared to DIC measured strain field at failure.

As shown in the previous strain analysis, the peaks appearing in the strain profile provide a direct method to determine the crack locations. Figure 8 shows the strain distribution obtained from DFOS at the bottom of the precast segmental beam at two different load levels. The two-dimensional (2D) strain field, obtained by DIC at the final failure stage, is also added as a reference of the real crack pattern in the concrete surface. In the DIC strain field at failure, various cracks are observed on the concrete surface, including oblique cracks in the bending–shearing zone and vertical cracks in the pure bending zone. Within the DIC measurement area, DFOS can identify a large number of those cracks that are about to occur at a very early stage, but DIC cannot yet identify them at this low load level. Some cracks are already very obvious in the DFOS profiles, at 25% of the ultimate load, as indicated by the vertical dashed lines. The position of the crack obtained by DFOS matches well with the DIC measurement, with an error in the crack location less than 1 cm, which is the spatial resolution adopted in the test.

### 3.2. Cross Section Strain Analysis of Cap Beam Damage

**Figure 9:**
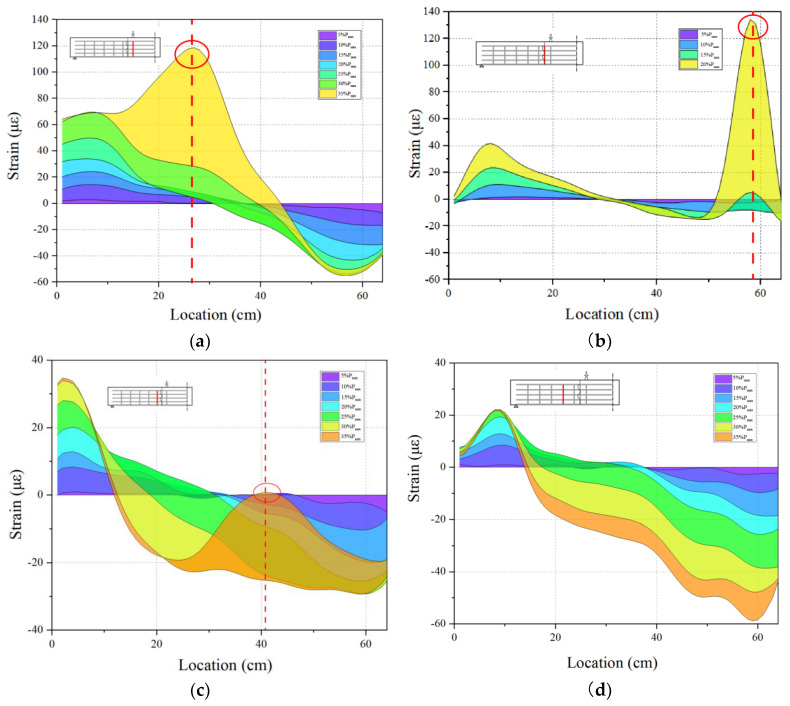
Strain profile, along cross-section depth, under ascending load for the segmental beam cap: (**a**) Section 1 (7 in Figure 4); (**b**) Section 2 (6 in Figure 4); (**c**) Section 3 (5 in Figure 4); (**d**) Section 4 (4 in Figure 4).

**Figure 10:**
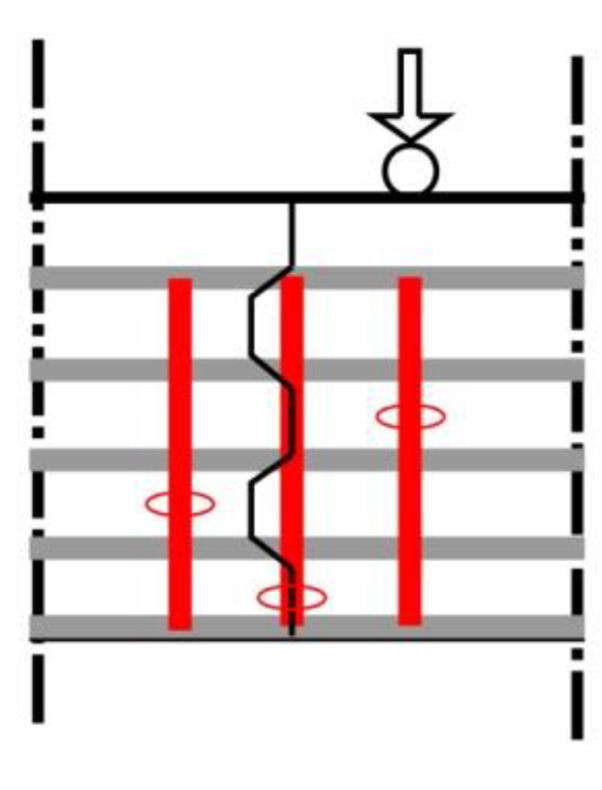
Location of cracks close to the shear keys in the segmental beam.

An improved evaluation of the beam damage was achieved through the analysis of the strain distribution across the section depth. The test focused on monitoring the cross-sectional strain state of the assembled segments, as shown in Figure 9. It can be observed, from cross-Section 1 at the middle segment (Figure 9a), that the strain exhibited linear elasticity characteristics at early loads, and the strain distribution changed significantly at 35% of the maximum load. A significant peak appeared in the strain, at a distance of about 27 cm from the top of the fiber line, indicating the occurrence of an extended crack at this position. In the cross-Section 2 at the shear key (Figure 9b), a significant peak appeared in the strain profile at 20% of Pmax, and the peak significantly increased thereafter. The peak was located at the bottom of the shear key, indicating the separation of the interface between segments, resulting in a crack. This marks the beginning of damage accumulation. Cross-Section 3, near the shear key, exhibited linear behavior at an early stage (Figure 9c), but as the load increased, the neutral axis position of the strain distribution continuously moved towards the top of the beam. When reaching 35% of Pmax, a significant peak appeared, indicating the occurrence of a crack in the lower part of the beam. As the cross section moved away from the shear key, the measured strain value decreased continuously, and although the neutral axis continued to move up with the load, there was no strain peak (as shown in Figure 9d). This indicates that, for the segmental beam, although the damage appeared earlier, the damage was always concentrated near the shear key, and its main damage came from the cracks developed near the shear key (as shown in Figure 10).

### 3.3. Strain Analysis in the Longitudinal Direction

**Figure 11:**
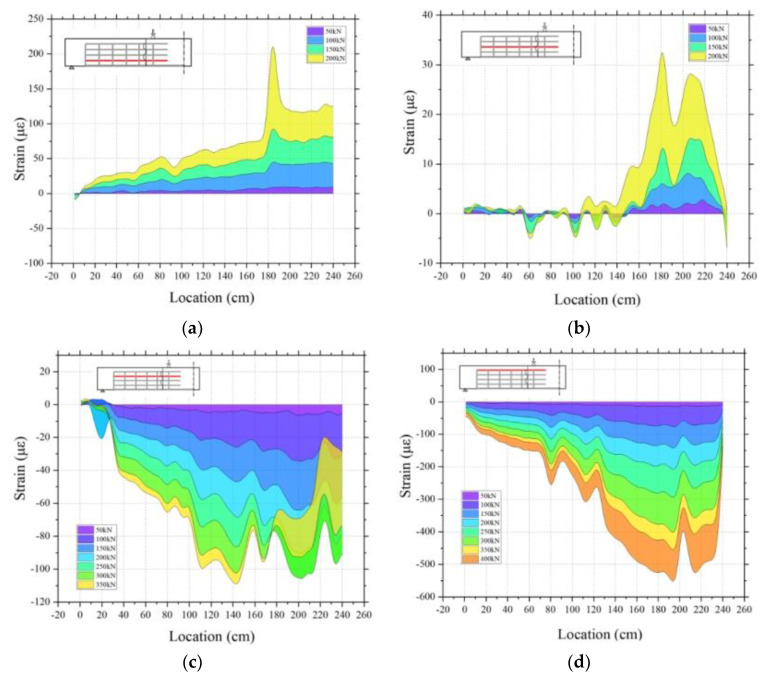
Longitudinal strain profiles at different cross-section depths for the segmental beam. (**a**) 16 cm from the bottom, (**b**) 32 cm, (**c**) 48 cm, (**d**) 64 cm.

The strain profile, along the longitudinal direction at different cross-section depths in the precast segmental beam, was also a focus of the test, as shown in Figure 11. From the longitudinal strain distribution of the segmental PSCBC beam, it can be seen that the test results follow the expected behavior predicted by classical elastic beam theory at low levels of load. The DFOS strain value at the bottom is the largest, and it decreases proportionally with the distance to the neutral axis. The strain value of the top fiber in the compression zone of the section becomes negative. At the same time, the development of cracks at different load stages is observed at different heights of the section, as shown in Figure 11a–d. A significant strain peak was found at the shear key position (Figure 11a), indicating the presence of cracks at the keyed joint. This peak was formed in the early stage of loading and continued to develop as the load increased. In addition, in the compression zone, the strain at the shear key crack appears as a strain valley, which is due to the complex stress state and cracking around the shear key, where the hypothesis of beam theory is no longer valid, and it is derived from a reduction in the compressive strain at this location. From the four different longitudinal strain profiles, it appears that the strain concentration at the shear key occurs before the 20% of Pmax. After 30% of Pmax, the strain mutation caused by cracks is observed clearly in all four longitudinal strain profiles. When the load reached 30% of Pmax, the crack length had already exceeded the third longitudinal section, and the height of the tension zone at the shear key had exceeded 56% of the section height.

### 3.4. Analysis of Failure Mode at the Shear Key 

**Figure 12:**
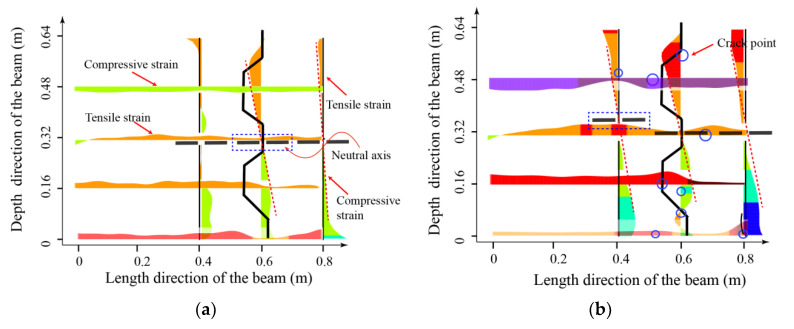

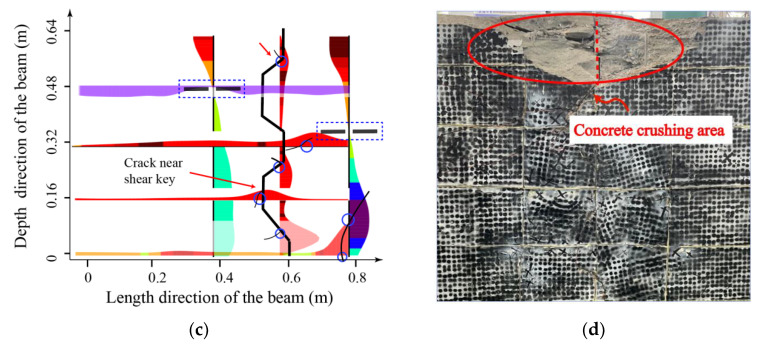
Evolution of shear key cracking at different damage levels and failure modes of the segmental beam: (**a**) elastic state; (**b**) elastoplastic state; (**c**) plastic state; (**d**) concrete crushing at the top of the joint.

The evolution of strain before cracking and the appearance of cracks close to the shear keys can be detected, located, and quantified by DFOS data, as presented in Figure 12. As the load increases, the detection, location, and progression of one or more cracks are indicated by bold dots. Once the location of the shear crack point is determined, the cracking pattern at the shear key under increasing load can be obtained, as shown in Figure 12, which provides a complete description of the shear crack development using DFOS.

It can be observed that, in the elastic stage (Figure 12a), the longitudinal strain distribution close to the shear key conforms to the deformation in the bending of a beam. The upper part of the section is in compression, and the lower part is in tension, with the neutral axis close to the middle of the cross-section. The strain on the shear key section roughly presents a linear distribution. The vertical strain along the cross-section depth in the shear key section shows tensile strain at the upper part of the beam and compressive strain at the lower part. As the load increases (Figure 12b), strain concentration areas appear around the shear key. These areas mainly concentrate near the key tooth. Among them, obvious strain peaks appear at the edge of the negative key tooth and at the corner of the positive key tooth. The neutral axis of the left segment tends to rise, while the neutral axis position of the middle segment shows no obvious change. With further increases in load (Figure 12c), the area with large gradient strain begins to develop macroscopic cracks. The cracks in the tension zone of the shear key expand, resulting in additional cracks. At the same time, it is worth noting that, at this point, the neutral axis position of the middle segment only slightly rises due to the expansion of the cracks on the tension side, while the neutral axis of the left segment rises significantly. The height of the tension zone in the figure reaches three times the height of the compression zone. This indicates that, with the cracking of the shear key, there has been a significant change in the load transfer path between segments. The zone at the middle of the segment remains relatively stable, while the sections passing through the shear key have large parts under tension. This is due to the lack of contact between the interfaces and the high reduction in the interaction force between segments provided by the cracked shear key, resulting, at the end, in the compressive failure at the top of the beam (as shown in Figure 12d).

**Figure 13:**
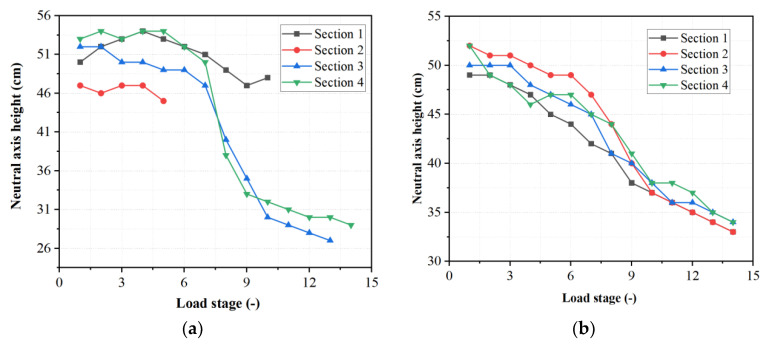
Neutral axis height variation under different loads: (**a**) PSCBC; (**b**) CICBC.

The evolution of the neutral axis location with increasing load levels, in both the PSCBC and CICBC beams, was also analyzed and shown in Figure 13. The height of the Y-axis in the figure refers to the distance from the neutral axis to the top of the beam. The overall height of the beam is 80 cm. Due to the prestressing force, the neutral axis is initially located below the middle of the beam height, which is about 50 cm from the top of the beam and 30 cm from the bottom. As the value of the ordinate decreases, it indicates that the neutral axis approaches the top of the beam. Due to the interference of cracks near the shear key, it was difficult to identify the neutral axis development for Section 1 and Section 2 in the PSCBC beam in the later stages. It can be observed from the figure that the rate of increase in neutral axis height in the PSCBC is significantly higher than that in the CICBC, indicating that the damage in the PSCBC beam is more severe. Moreover, the changes in the neutral axis height, between different sections of the PSCBC, exhibit some inconsistencies, while those in the CICBC beam are relatively consistent with what is expected from beam theory: the variation of the neutral axis position is proportional to the load increment. This, again, confirms that the beam theory does not apply in the zones with discontinuities and complex stress states, as is the case of the zones close to the shear keys in the PSCBC cap beam.

## 4. Conclusions

This paper investigates the potential of using DFOS technology to study the damage characteristics of two types of bent cap beams of bridges: either made of precast segments or cast in situ. There were two types of optical fiber cables used to explore the applicability of different types of optical fibers in monitoring concrete segmental bridges. Optical fiber strain sensors were bonded on the concrete surface of multiple cross-sections and longitudinal sections of the beams. The tests measured the response, damage process, and failure modes of both types of bent cap. The main findings of this study are as follows:

1. DFOS have the capability to capture surface strains on prestressed segmental bent cap components. The changes in strain can quantitatively reflect the variation of load carrying capacity and the development of cracks in the cap beam. The peak and valley values of strain detected by DFOS can accurately determine the location of crack development, even before visible cracks appear to the naked eye. This is not only demonstrated for the cast-in-situ element, as experienced in other research works, but also for the precast segmental elements for the first time in the present study.

2. Looking at the different types of optical fibers for damage-monitoring applications in segmental concrete bridges, polyimide optical fibers show high sensitivity and are more suitable for the detection of sudden changes in local strains. However, polyimide is brittle and should be better protected during the construction process and operating phase. They are also prone to the appearance of strain-reading anomalies (spikes) at high levels of strain. Multi-coated optical fibers have stronger environmental adaptability, but their diameter is large, which may pose some difficulties in their deployment. In addition, the strain data of multi-coated optical fibers fluctuate more smoothly, which may mask some local crack information. These results confirm the conclusions of previous experimental works on monolithic concrete beams [17,21,36].

3. DFOS can be applied to measure the overall strain of the large-scale beam, and the data can reflect the damage characteristics of each section. By analyzing the strain data of the cross-section, it is possible to determine the height of the neutral axis and evaluate the damage state of the beam in a quantitative manner. The optical fiber strain distribution in the longitudinal direction reflects the distribution of cracks along the span, and the local strains that are greater than the average strain in the distributed strain profile can be used to quantify the crack propagation.

4. DFOS monitoring has allowed for the confirmation that the damage process and characteristics of precast segmental concrete members are significantly different from cast-in-place elements. The stress state on the shear key interface of the segmental deck is complex, and DFOS deployed in a grid arrangement close to the key were able to follow this complex distribution. As a weak point of the segmental deck, the presence of the shear key causes changes in the stress state along the length of the member. The crack-propagation rate of the segmental concrete element is higher than that of the cast-in-place. As the crack on the shear key forms, the damage of the segmental part accumulates continuously, and the expansion of the crack leads to the premature formation of a concentrated compression zone in the upper part of the shear key. This may eventually lead to the failure under compression of the concrete at the shear key.

In summary, the data obtained from the DFOS system can inform the design and construction of future prestressed cap beams or other bridge elements, such as the deck, which is made of precast units. It also validates the feasibility of using DFOS technology not only in the monitoring of monolithic concrete elements but also for segmental built elements with the presence of shear key joints. In addition, the results are of great value to develop strategies for the monitoring and preventive maintenance of existing precast segmental bridges.

## Figures and Tables

**Table 1:** Mean value and variance of strain increments from different optical fibers (με).

Fiber Type	V1		J1	
Load Level	Mean (με)	Var (με^2^)	Mean (με)	Var (με^2^)
5%	0.056	4.12	0.196	164.84
15%	0.183	3.47	0.404	1469.42
25%	0.457	3.70	0.499	1469.51
35%	0.922	6.23	0.596	3284.53
45%	1.081	8.73	0.548	13,744.96
55%	2.75	27.78	0.452	13,609.21

## Data Availability

Data is unavailable due to privacy or ethical restrictions.
